# Implication of Polyhistidine, a Novel Apoptosis Inhibitor, in Inhibiting Lipopolysaccharide-Induced Apoptosis in Boar Sperm

**DOI:** 10.3390/ani9100719

**Published:** 2019-09-24

**Authors:** Tianzeng Song, Yi Shi, Yangang Wang, Izhar Hyder Qazi, Christiana Angel, Ming Zhang

**Affiliations:** 1 College of Animal Science & Technology, Sichuan Agricultural University, Chengdu 611130, China; songtianzeng@china.com.cn (S.T.); shiyi9005@163.com (Y.S.); idsuperwang@sina.com (Y.W.); vetdr_izhar@yahoo.com (I.H.Q.); 2 Institute of Animal Science, Tibet Academy of Agricultural and Animal Husbandry Science, Lhasa 850009, China; 3 Department of Veterinary Anatomy & Histology, Shaheed Benazir Bhutto University of Veterinary and Animal Sciences, Sakrand 67210, Pakistan; 4 Department of Veterinary Parasitology, College of Veterinary Medicine, Sichuan Agricultural University, Chengdu 611130, China; qazi5502@yahoo.com; 5 Department of Veterinary Parasitology, Faculty of Veterinary Sciences, Shaheed Benazir Bhutto University of Veterinary and Animal Sciences, Sakrand 67210, Pakistan

**Keywords:** apoptosis, boar, lipopolysaccharide, polyhistidine (pHis), sperm

## Abstract

**Simple Summary:**

Gram-negative bacteria are the main pathogenic microorganisms found in human and animal semen. Lipopolysaccharide (LPS), a component of the cell wall of Gram-negative bacteria, has been linked to inducing apoptosis in human and rat sperm; however, little is known regarding LPS-induced apoptosis in boar sperm. This detrimental effect of LPS is potentially mediated via competitive bidding with toll-like receptor (TLR) 4 on the cytoplasmic membrane. Therefore, it is reasonable to elucidate the potential mechanisms by which the binding of LPS and TLR4 could be prevented. Polyhistidine is widely used for the delivery of nucleic acids and antibodies into the cell cytoplasm, and it is a novel TLR4 agonist. In the current study, we envisaged that pHis might also serve as an effective tool for inhibiting LPS-induced apoptosis in boar sperm. The new finding of our present study is that pHis could inhibit, to some extent, LPS-induced boar sperm apoptosis, and it could ameliorate the overall sperm quality parameters under liquid storage or at 37 °C incubation conditions. However, further investigation should be continued to fully elucidate the mechanistic basis of these ameliorative effects of pHis.

**Abstract:**

Lipopolysaccharide (LPS) released from Gram-negative bacteria binds to toll-like receptor 4 (TLR4) and induces boar sperm apoptosis. Similarly, polyhistidine (pHis), a TLR4 agonist, can also bind to TLR4. We hypothesized that pHis could inhibit LPS-induced sperm apoptosis by competitively binding to TLR4 to then improve sperm quality. Therefore, the objective of this study was to examine whether pHis can inhibit LPS-induced sperm apoptosis and affect sperm quality. The results showed that the concentrations of bacterial colonies were significantly increased from 36 to 120 h under liquid storage conditions (*p* < 0.05); however, concentrations of LPS in boar semen showed a relatively constant trend (4.98 ± 1.55 EU/mL) following 120 h storage. The addition of 100 μg/mL pHis in the BTS extender significantly improved boar sperm motility and viability at 37 °C, and it significantly (*p* < 0.05) inhibited boar sperm apoptosis under liquid storage (17 °C) and at 37 °C incubation conditions. The co-treatment of LPS and pHis further confirmed that pHis played its role in inhibiting LPS-induced sperm apoptosis. In conclusion, our preliminary findings provide reasonable evidence that pHis could act as an inhibitor of LPS-induced apoptosis in boar sperm stored for longer periods of time. pHis might inhibit LPS-induced sperm apoptosis by competitively binding to TLR4. Nevertheless, further mechanistic studies are awaited to fully elucidate its potential implication in inhibiting LSP-induced apoptosis.

## 1. Introduction

Bacterial contamination in boar semen is inevitable, largely because the routine procedures of semen collection and manipulation are difficult and generally fail to provide controlled and aseptic ejaculate collections [1,2,3]. It has been demonstrated that Gram-negative bacteria are the most prevalent type of pathogenic microorganism that contaminates the semen [4,5,6,7]. Generally, Gram-negative bacteria have been implicated in altering the pH, sperm metabolic activities, and inducing excessive production of free radicals in seminal plasma and, as a result, impairing the sperm quality and overall fertility [8]. Besides, Gram-negative bacteria also release endotoxins such as lipopolysaccharide (LPS) during bacteriolysis, and they consequently induce sperm apoptosis [9,10] and immune responses in the female genital tract [11,12,13]. Previously, it has been reported that certain antibiotics or chemicals when added to the semen extender could reduce bacteria-induced damage to the sperm and can possibly inhibit bacterial proliferation [14,15]. However, these antibiotics or chemicals are not able to clear the LPS released from Gram-negative bacteria during bacteriolysis [16].

At present, it is well established that LPS in seminal plasma could induce sperm apoptosis, and it may result in further decline in the overall quality of the ejaculate [17,18,19,20]. The apoptosis signaling pathway is activated by intracellular adaptors, which are triggered and recruited by the binding of LPS and toll-like receptor (TLR) 4 on the cytoplasmic membrane [21]. Therefore, for preventing the onset of LPS-induced sperm apoptosis, it seems reasonable to develop and investigate the possible mechanisms by which the binding of LPS and TLR4 could be prevented. It is worthwhile to mention that binding of LPS and TLR4 largely depends on certain molecular determinants such as cluster of differentiation-14 (CD14), myeloid differentiation protein 2 (MD2), and LPS-binding protein (LBP) [22]. Mounting this evidence, it is apparent that LPS-induced sperm apoptosis could be inhibited by preventing/attenuating the endotoxic activity of LPS [17], certain antagonists or agonists such as chitosan (LPS structural analog) [23], or eliminating CD14, MD2, or LBP [24,25,26,27].

Polyhistidine (pHis) is widely used for the delivery of nucleic acids and antibodies into the cell cytoplasm [28]. Six polyhistidine residues are commonly used in immobilized metal-affinity chromatography (IMAC) to purify recombinant proteins, and polyhistidine in metal cation solution exhibits cationic amphiphilicity, which is characteristic of small molecules CD14/TLR4/MD-2 [29]. Lipid A of LPS enables its interaction with a wide variety of cationic amphiphiles such as small peptides, amine dendrimers, pentamidines, polyamines, or gemini surfactants. Optimization in the design of cationic amphiphiles allows the maximization LPS affinity [30]. Therefore, it is possible that it is an alternative to the conventional TLR4 agonist, by binding to TLR4, or antagonist, by binding to lipid A of LPS. Polymyxin B (PMB), a polycationic antibiotic, is known to interact with the LPS complex and neutralize its endotoxic activity; therefore, PMB is often used a positive control for eliminating the possible impact of LPS on boar sperm in some studies [31,32].Therefore, we speculated that pHis might also serve as an effective tool for preventing the binding of LPS and TLR4 and thereby inhibiting the LPS-induced apoptosis in boar sperm. Therefore, in this study, we evaluated the potential implication of pHis in preventing LPS-induced apoptosis in boar sperm and whether pHis affected semen quality.

## 2. Materials and Methods

### 2.1. Chemicals and Extender

Polymyxin B (PMB) was purchased from Amresco (Amresco; St. Solon, OH, USA). Glucose, xodium citrate, ethylenediamine tetraacetic acid (EDTA), sodium bicarbonate (NaHCO_3_), potassium chloride (KCl), LPS, and pHis were purchased from Sigma chemicals Co (Sigma; St. Louis, MO, USA).

### 2.2. Semen Collection and Preparation

Sperm-rich fractions of ejaculates were collected from sexually mature boars (*n* = 25 boars), aged 16–30 months, using the gloved-hand technique. Only ejaculates with sperm viability ≥80% and sperm motility ≥75% were used for the subsequent experiments. In order to get pooled samples, semen from 5 boars was mixed to get a single pooled sample; thus, samples from all boars constituted a total of 5 pooled samples (*n* = 5). The semen samples were diluted with BTS extender (Beltsville Thawing Solution: 3.7 g of glucose, 0.3 g of Na_3_ citrate, 0.125 g of NaHCO_3_, 0.125 g of Na_2_-EDTA, 0.075 g of KCl, 0.6 g/L penicillin G sodium, and 1.0 g/L dihydrostreptomycin, all diluted to 100 mL) to a concentration of 4 × 10^6^ sperm/mL and kept at 17 °C (liquid storage temperature) up to 5 d or incubated at 37 °C for 24 h. The BTS extender was prepared as described previously [33].

### 2.3. Experimental Design

In experiment 1, semen samples were diluted in BTS extender containing 1000 IU/mL of penicillin G and 1000 μg/mL of streptomycin. Changes in the bacterial colonies and LPS concentrations were measured at 0, 6, 12, 36, 72, and 120 h under liquid storage conditions.

In experiment 2, potential implications of pHis on semen quality were assessed. Sperm were incubated in BTS extender with or without 100 μg/mL of pHis (Cas No.26062-48-6, Molecular weight: 5000–25000 Da, Sigma) at 37 °C. Meanwhile, BTS extender containing 100 μg/mL of PMB (Amresco; St. Solon, OH, USA) was used as a positive control. Semen quality (sperm motility, movement speed) and the sperm apoptosis rate were examined at 0, 1, 3, 6, 9, 12, and 24 h at 37 °C incubation and at 0, 6, 12, 36, 72, and 120 h under liquid storage conditions after pHis or PMB co-incubation.

In experiment 1, LPS was detected in boar semen. Whereas, in experiment 2, it was observed that pHis not only inhibited sperm apoptosis but also improved the semen quality. Therefore, based on these findings, we hypothesized that pHis is implicated in reducing sperm apoptosis via blocking LPS-TLR4. Therefore, experiment 3 was designed. Herein, sperm was preincubated in BTS extender with 100 μg/mL of pHis for 30 min, and then 10 μg/mL of LPS was added into the BTS extender to confirm the potential implication of pHis in reducing the rate of sperm apoptosis following LPS stimulation.

In these experiments, 100 μg/mL of pHis and 10 μg/mL of LPS were determined by a preliminary experiment in our laboratory (Figure S1).

### 2.4. Quantification of Bacterial Colonies

Aerobic cultures (6.9% CO_2_) were performed on all samples at 37 °C for 24 h using agar plate culture medium containing 0.5% beef extract, 1% peptone, 0.5% NaCl, and 1.5% agar. The bacterial colonies were counted, and the concentration of bacterium in each sample was calculated [23].

### 2.5. Detection of LPS in Semen by Limulus Assay

The concentration of LPS in seminal plasma was measured by the limulus assay [34] using a limulus chromogenic substrate kit (Pyrochome, Associate of Cape Cod, MA, USA). Briefly, samples were diluted with endotoxin-free distilled water and then heated at 70 °C for 10 min. After centrifugation at 1500 rpm for 10 min, the supernatant was mixed with limulus amebocyte lysate for up to 120 min at 30 °C. The optical density (OD) of reaction was detected by Varioskan (Thermo, NY, USA) at 545 nm wavelength. The level of LPS was calculated using the pre-established regression of LPS concentration and OD.

### 2.6. Semen Quality Assessment

Routine sperm quality parameters such as, sperm viability, motility, and movement speed were measured. The sperm membrane status was analyzed using a SYBR-14 and propidium iodide (PI) sperm viability kit (Invitrogen, CA, USA) according to the manufacturer’s instructions. The fluorescent staining of sperm was observed and photographed using fluorescence microscopy (Nikon 90i, Tokyo, Japan). Sperm motility and movement speed were assessed using a fully automated sperm quality analyzer (Medical electronic system Co., Sperm Quality Analyzer (SQA)-V Gold, MES, Israel) according to the manufacturer’s guidelines as described in the operation manual. Firstly, the concentration of sperm in semen samples was adjusted to about 20 million. Then, the semen samples were loaded in a measurement capillary and inserted into an automated measurement compartment of SQA-V. The sperm motility index (SMI) and average path velocity (VAP) were automatically test.

### 2.7. Detection of Sperm Apoptosis

Early apoptosis of sperm was examined using an Annexin V-FITC Apoptosis Kit (ApoAlert^®^ Annexin V-FITC Apoptosis Kit, KeyGen BioTech, Wuhan, China) as per the manufacturer’s instructions. Briefly, 5 × 10^6^ sperm were resuspended in 500 μL of binding buffer (ready to use) and mixed with 5 μL Annexin V–FITC and 5 μL PI, and then the mixture was incubated at room temperature for 15 min in darkness. Fluorescence staining of sperm was monitored by fluorescence microscopy (Nikon 90i, Tokyo, Japan). Apoptotic sperm were recognized, and the early apoptosis rate was calculated using Image Pro Plus 6.0 software (Media Cybernetics, MD, USA) according to the following formula: Apoptosis percentage = $\frac{\mathrm{Number}\;\mathrm{of}\;\mathrm{sperm}\;\mathrm{with}\;\mathrm{FITC}}{\mathrm{Total}\;\mathrm{number}\;\mathrm{of}\;\mathrm{sperm}}\times 100\%$ [35].

### 2.8. Statistical Analysis

Data were analyzed using IBM SSPS statistics v17.0 (IBM statistics, NY, USA). Data obtained from five pooled samples are presented as mean ± SE. All percentage data were subjected to arcsine square-root transform before performing ANOVA. A Duncan’s test was used to analyze the data for significant differences. *p* < 0.05 was considered statistically significant.

## 3. Results and Discussion

### 3.1. Changes in Concentration of Bacterial Colonies and LPS Under Liquid Storage Conditions

**Figure 1:**
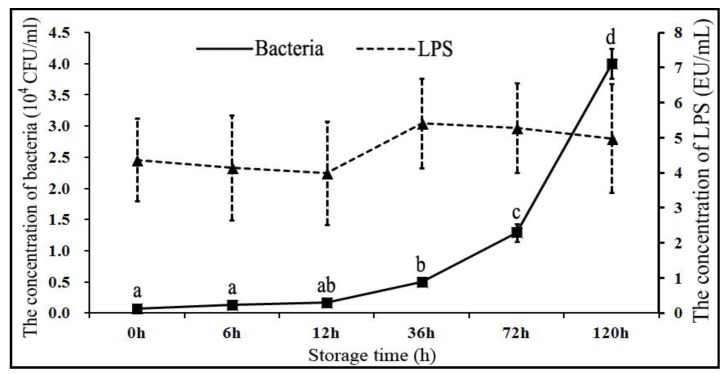
Changes in the concentration of bacterial colonies and lipopolysaccharide (LPS) in boar semen stored at 17 °C (liquid storage conditions). The values are presented as mean ± SE (*n* = 5 pooled samples). ^a, b, c, d^ indicate significant differences (*p* < 0.05).

The changes in bacterial and LPS concentrations at different time points are presented in Figure 1. Briefly, following the storage of boar sperm in BTS extender at 17 °C for 120 h, the bacterial concentration in semen was significantly increased (*p* < 0.05) from 4.98 ± 0.38 × 10^3^ CFU/mL (36 h), to 12.92 ± 1.42 × 10^3^ CFU/mL (72 h), and to 40.02 ± 2.38 × 10^3^ CFU/mL (120 h). These changes in bacterial concentration indicated that the bacterial colonies continued to grow rapidly after 36 h storage, despite the presence of 1000 IU/mL of penicillin G and 1000 μg/mL of streptomycin in BTS extender. Previous studies have also demonstrated that boar semen was contaminated with certain bacteria such as *Escherichia coli* and *Pseudomonas aeruginosa* in more than 80% of the boars [4,5,6,7]. It has been reported that the usual bacterial concentration in fresh semen is around 10^3^–10^5^ CFU/mL [35,36]. However, the increase in bacterial concentration in our study was strikingly very high and elicited the potential impact of storage time on overall sperm quality. Over the years, contamination of boar semen with Gram-negative bacteria has remained a major concern in the swine artificial insemination industry [2,3,5], and its potential consequences with regard to reduced sperm quality have also been at the center of attention. Conventional antibiotics (such as penicillin G and streptomycin) could only weaken the cell wall or suppress the protein synthesis in bacteria, and they are not fully capable of clearing the bacterial growth in semen [14,15,16]. Other measures have to be adopted to overcome this practical problem.

Intriguingly, in our study, the LPS concentration in semen did not change significantly with an increasing storage time (Figure 1). It was observed that the Gram-negative bacteria were the major contaminants in the boar semen, usually more than 80% of the total number of bacteria [2,3,5], and the bioactivity of LPS was 4.98 ± 1.55 EU/mL in semen. Theoretically, the LPS bioactivity in semen should have undergone a significant increase with bacterial proliferation, but it kept a constant bioactivity instead. In contrast to our findings, Okazaki et al. (2010) reported that treatment with penicillin G alone significantly increased the levels of LPS in boar semen [14]. Although, the exact basis for such discrepancy in observations is not currently known; however, one of the possible reasons is that, conventionally, LPS binds to the TLR4 on the boar sperm membrane and might have been utilized in eliciting sperm apoptosis with increasing semen storage time. Besides, LPS might have undergone spontaneous degradation in the BTS extender. Nevertheless, the exact underlying mechanisms for this dynamic equilibrium between LPS production and utilization in boar semen during liquid storage conditions are largely unclear and require further investigation in well-powered studies. Previously, it has been demonstrated that LPS binds to TLR4 and thereby triggers sperm apoptosis [10]. Based on our current findings and previous evidence, it is understood that LPS accumulates in the semen during storage, and it could induce sperm apoptosis [9,10]. Therefore, for inhibiting sperm apoptosis during liquid storage conditions, it is essential to counter LPS or block the potential interaction of LPS and TLR4.

### 3.2. Effects of pHis on Boar Sperm Quality

**Figure 2:**
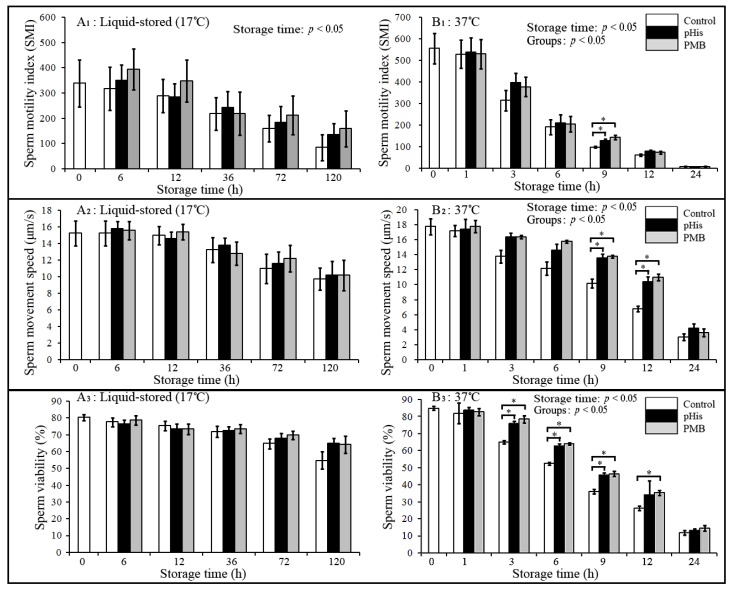
Changes in the sperm motility index, movement speed, and viability during liquid storage and at 37 °C incubation. * indicates a significant difference between the groups (*p* < 0.05). Panels A_1_, A_2_, and A_3_ depict the changes in sperm motility, movement speed, and viability, respectively, in liquid-stored (17 °C) boar semen; whereas panels B_1_, B_2_, and B_3_ depict changes in sperm motility, movement speed, and viability, respectively, in boar semen incubated at 37 °C. Boar sperm were diluted with BTS extender in the control group, and 100 μg/mL PMB or pHis was added to the BTS extender in PMB or pHis-treated groups. The changes in the sperm motility, movement, and viability were correlated with storage time extension in 37 °C incubation groups.

The results of changes in the sperm quality parameters such as sperm motility index (SMI), movement speed, and viability in pHis, PMB, and the control group are depicted in Figure 2. Briefly, boar SMI, movement speed, and viability at 37 °C showed a significant decease with increasing storage time (storage time: *p* < 0.05); however, no significant difference was observed between pHis, PMB, and control groups under liquid storage conditions (17 °C). SMI in pHis and PMB groups was significantly higher compared to the control group at 9 h postincubation at 37 °C, and sperm movement speed in pHis and PMB groups was significantly higher compared to the control group at 9 and 12 h post-incubation at 37 °C. Sperm viability in pHis and PMB groups was significantly higher compared to the control group from 3 to 9 h post-incubation at 37 °C. These results suggest that pHis and PMB (the positive control) could significantly improve the quality of boar sperm at 37 °C. PMB binds to the LPS complex and is able neutralize LPS endotoxic activity [32], and it has also been reported that treatment with PMB could increase sperm motility and membrane integrity in frozen-thawed sperm [14]. Therefore, based on our results and past evidence [14,31,32], it reasonable to assume that PMB could improve the quality of boar sperm because it performs dual roles (i.e., it could suppress bacterial proliferation and might also block the interaction of LPS and TLR4). Meanwhile, in the present study, we also report a novel finding with regards to the potential role of pHis in improving the quality of boar sperm.

### 3.3. Implication of Polyhistidine in Inhibiting Sperm Apoptosis

**Figure 3:**
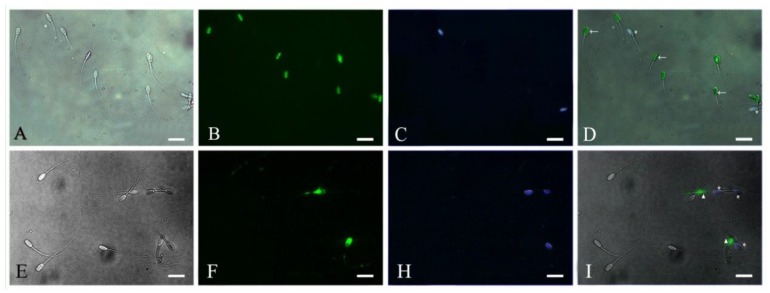
Photomicrographs of early apoptotic and dead sperm. A and E: Light-field phase contrast microscopic image. B: Live sperm with SYBR-14. C and H: Dead sperm with propidium iodide (PI). D: Merge of A, B, and C. F: Early apoptotic sperm with FITC. I: Merge of E, F, and H. Scale bar: 20μm. ←: the live sperm. *: the dead sperm. ▲: the early apoptotic sperm.

**Figure 4:**
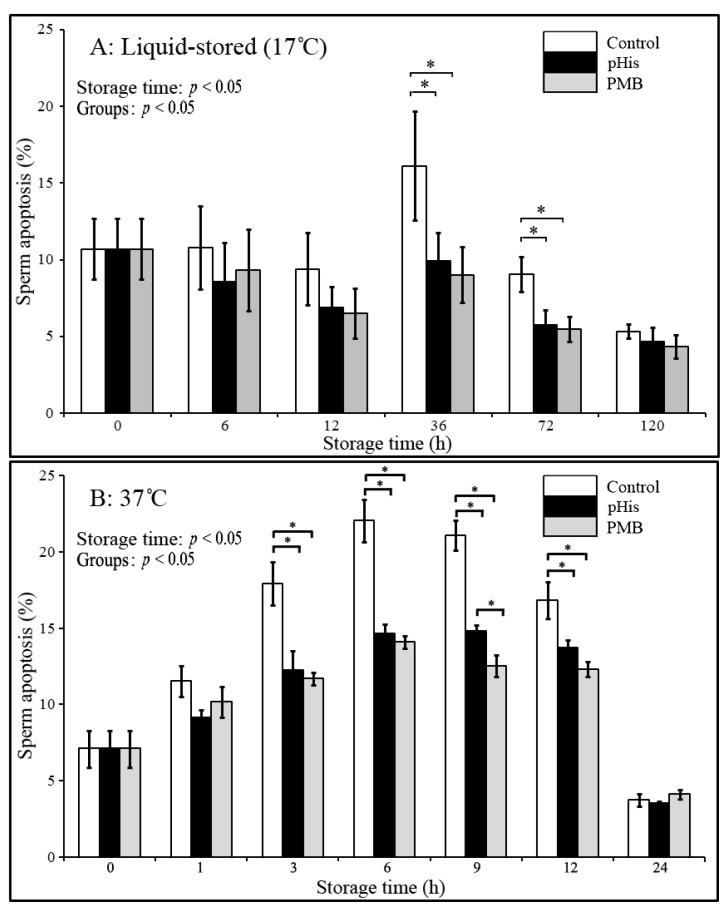
Changes in sperm apoptosis during liquid storage (17 °C) and at 37 °C incubation. * indicates a significant difference (*p* < 0.05). Boar sperm were diluted with BTS extender in the control group, and 100 μg/mL PMB or pHis was added in the BTS extender in PMB or pHis groups.

Based on evidence from the above results, in the next experiment we evaluated the potential implication of pHis in inhibiting/reducing the rate of apoptosis in boar sperm in two different storage conditions. To this effect, boar sperm were dyed with different fluorescent labels for detecting the early apoptotic and dead sperm (Figure 3) under both liquid storage conditions and at 37 °C (Figure 4). The results showed that sperm apoptosis in pHis and PMB groups was significantly lower (*p* < 0.05) compared to the control group at 36 and 72 h under liquid storage and at 3, 6, 9, and 12 h at 37 °C incubation conditions. These findings indicate that both pHis and PMB have significant implications in inhibiting sperm apoptosis under both liquid storage and 37 °C incubation conditions. Previous studies have shown that bacterial contamination and LPS caused apoptosis in humans [9,10] and boar sperm [19]. Therefore, as evidenced from the present study, it is reasonable to envisage that LPS and proliferating bacteria induced apoptosis in boar sperm, and the treatment of sperm with pHis and PMB could reduce the rate of apoptosis during boar semen storage for longer periods of time. It is believed that PMB could inhibit sperm apoptosis because it could suppress the bacterial proliferation and prevent LPS binding to TLR4 [14]. The binding of LPS and TLR4 leads to the activation of the apoptosis signaling pathway in immune cells (or in some somatic cells) [32] and human sperm [14]. Polyhistidine could reduce the LPS-triggered onset and level of apoptosis in sperm.

### 3.4. pHis Inhibited LPS-Induced Sperm Apoptosis

**Figure 5:**
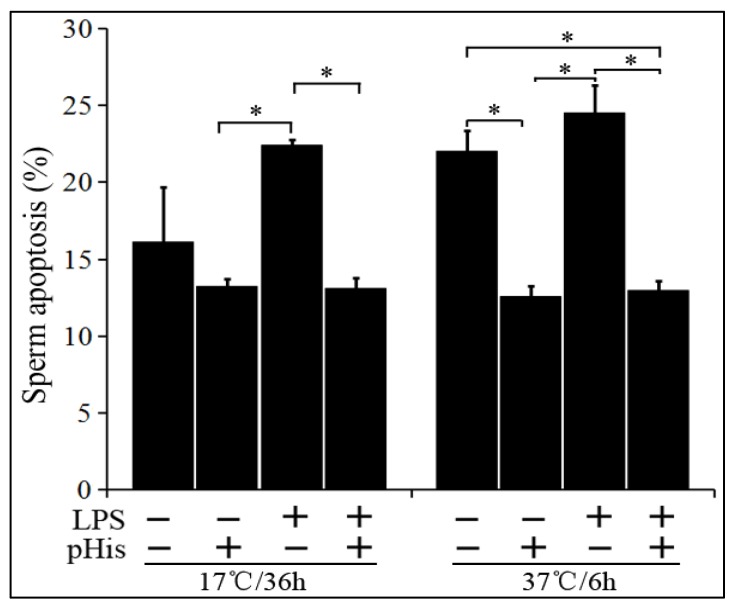
pHis inhibited LPS-induced sperm apoptosis in both liquid-stored (17 °C) and incubated boar semen (37 °C).* indicates a significant difference (*p* < 0.05).

In the next phase of the experiment, we assessed the effect of pHis pre-treatment on LPS-induced boar sperm apoptosis. The boar sperm was treated with LPS alone or co-treated with LPS and pHis (pre-treatment). It was stored at 17 °C for 36 h or at 37 °C for 6 h, and the rate of sperm apoptosis was examined (Figure 5). The results showed that LPS, when incubated alone, significantly (*p* < 0.05) induced apoptosis in boar sperm at both liquid storage (17 °C) and 37 °C incubation conditions. However, LPS could not induce significant apoptosis in boar sperm when the semen was preincubated with 100 μg/mL of pHis for 30 min. These observations suggest that addition of pHis significantly (*p* < 0.05) inhibited LPS-induced boar sperm apoptosis. 

## 4. Conclusions

In summary, the new finding of our present study is that pHis could inhibit, to some extent, boar sperm apoptosis induced by LPS, and it could improve the overall sperm quality under liquid storage or at 37 °C incubation conditions. This effect of pHis could be attributable to its capability to competitively bind TLR4 and might serve as possible factor for preventing the conventional interaction of LPS and TLR4 during storage of boar semen contaminated with bacterial loads. Nevertheless, further well-powered and mechanistic studies are awaited to fully elucidate the fundamental basis for this potential implication of pHis in reducing the rate of apoptosis in the stored boar semen.

## Supplementary Materials

The following are available online at http://www.mdpi.com/2076-2615/9/10/719/s1, Figure S1: The preliminary experiment for determining the dosage LPS and pHis. A and C: Sperm apoptosis rate at 17 °C, B and D: Sperm apoptosis under 37 °C. In C and D, sperm was pre-incubated in different dosage of pHis for 30 min, and then added 10 μg/mL of LPS. a, b, c means the significant difference within the same time-point. m, n, p, q indicates the significant difference with time-course in 0 μg/mL of LPS (A and B) or 0 μg/mL of pHis (C and D). The results suggested that 10 μg/mL of LPS and 100 μg/mL of pHis is the appropriate dosage. 100 μg/mL of pHis and 10 μg/mL of LPS.
