# Supplementary material for: Implication of Polyhistidine, a Novel Apoptosis Inhibitor, in Inhibiting Lipopolysaccharide-Induced Apoptosis in Boar Sperm
Source: Animals (Basel). 2019 Sep 24;9(10):719. doi: 10.3390/ani9100719 (PMC6827019; doi:10.3390/ani9100719)
Supplement: Supplementary file 1 [file animals-09-00719-s001.pdf]

animals

Supplementary Materials: Implication of Polyhistidine, a Novel Apoptosis Inhibitor, in Inhibiting Lipopolysaccharide-Induced Apoptosis in Boar Sperm

Tianzeng Song [1,2], Yi Shi [1], Yangang Wang [1], Izhar Hyder Qazi [1,3], Christiana Angel [4,5], Ming Zhang [1,*]

1 College of Animal Science & Technology, Sichuan Agricultural University, Chengdu 611130, China; songtianzeng@china.com.cn (S.T.); shiyi9005@163.com (Y. S.); idsuperwang@sina.com (Y. W.); vetdr_izhar@yahoo.com (I.H.Q); zhangming@sicau.edu.cn (M. Z.)
2 Institute of Animal Science, Tibet Academy of Agricultural and Animal Husbandry Science, Lhasa 850009, China
3 Department of Veterinary Anatomy & Histology, Shaheed Benazir Bhutto University of Veterinary and Animal Sciences, Sakrand 67210, Pakistan
4 Department of Veterinary Parasitology, College of Veterinary Medicine, Sichuan Agricultural University, Chengdu 611130, China; qazi5502@yahoo.com
5 Department of Veterinary Parasitology, Faculty of Veterinary Sciences, Shaheed Benazir Bhutto University of Veterinary and Animal Sciences, Sakrand 67210, Pakistan
* Correspondence: zhangming@sicau.edu.cn

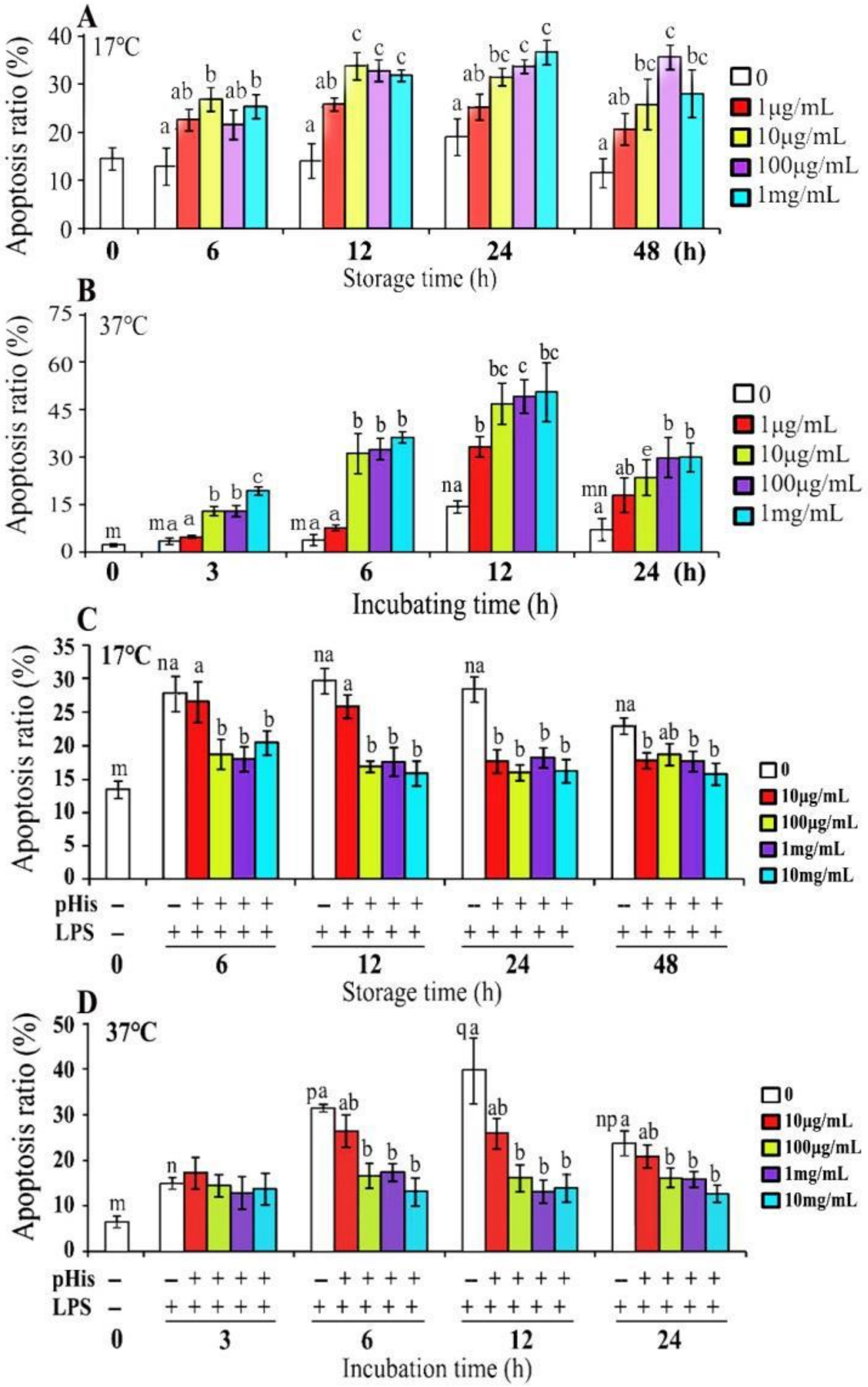

Figure S1. The preliminary experiment for determining the dosage Lipopolysaccharide (LPS) and Polyhistidine (pHis). A and C: Sperm apoptosis rate at 17 °C, B and D: Sperm apoptosis under 37 °C. In C and D, sperm was pre-incubated in different dosage of pHis for 30 min, and then added 10 µg/mL of LPS. a, b, c means the significant difference within the same time-point. m, n, p, q indicates the significant difference with time-course in 0 µg/mL of LPS (A and B) or 0 µg/mL of pHis (C and D). The results suggested that 10 µg/mL of LPS and 100 µg/mL of pHis is the appropriate dosage. 100 µg/mL of pHis and 10 µg/mL of LPS.
